# Diagnosis and Treatment of Esophageal Granular Cell Tumor: A Case Report and Review of the Literature

**DOI:** 10.1155/2017/1071623

**Published:** 2017-11-22

**Authors:** Ramin Niknam, Kamran Bagheri Lankarani, Bita Geramizadeh

**Affiliations:** ^1^Gastroenterohepatology Research Center, Shiraz University of Medical Sciences, Shiraz, Iran; ^2^Health Policy Research Center, Shiraz University of Medical Sciences, Shiraz, Iran; ^3^Department of Pathology, Transplant Research Center, Shiraz University of Medical Sciences, Shiraz, Iran

## Abstract

Gastrointestinal granular cell tumors are uncommon. The most common site of gastrointestinal granular cell tumor (GCT) is esophagus. We report a case of esophageal GCT incidentally diagnosed by endoscopy. The lesion was evaluated by endoscopic ultrasonography and resected using the endoscopic technique without complication.

## 1. Introduction

Granular cell tumor (GCT) was described in 1926 by Abrikossoff [1]. These tumors may arise in different organs [2]. The origin of GCTs is the Schwann cell. These tumors consist of fusiform and polygonal cells in compact nests with dark nuclei and abundant eosinophilic granular cytoplasm [3, 4].

The most common site of gastrointestinal GCTs is esophagus that only a few cases have been reported in the literature [4–6]. In this report, we described a case of esophageal GCT treated by the endoscopic technique. We also review some literature on diagnosis and management of GCTs.

## 2. Case Report

**Figure 1:**
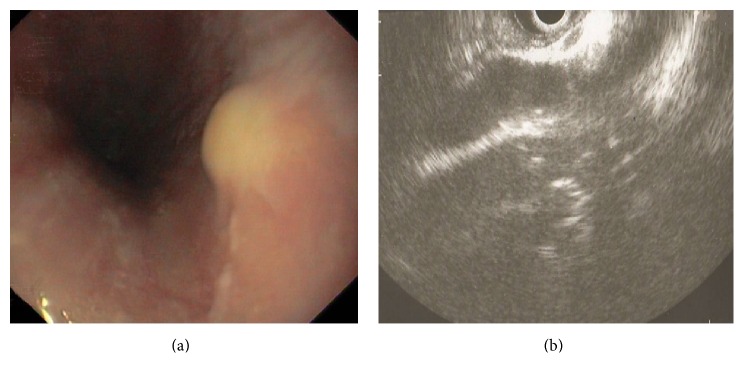
Endoscopic view of an elevated yellow submucosal lesion in esophagus (a). Endosonographic image showed a nonhomogeneous hyperechoic submucosal lesion with a diameter of 12 mm and without extension to muscularis layer (b).

A 50-year-old woman presented to our center with dyspepsia from three months prior to evaluation. Past medical history was unremarkable. The physical examination was normal. Upper gastrointestinal endoscopy was performed for evaluation of cause of dyspepsia. Endoscopy identified a firm submucosal yellow lesion, measuring approximately 10 mm and located 30 cm distal from the incisor teeth (Figure 1). The stomach and duodenum were normal. Biopsies by forceps were taken from the esophageal lesion. The histologic assessment was in favor of GCT.

Endoscopic ultrasonography (EUS) was performed by linear echo with frequency of 10 MHZ. EUS identified a submucosal lesion with a diameter of 12 mm. It was a nonhomogeneous hyperechoic solid lesion with clear border with no extension to muscularis layer and without mediastinal adenopathies (Figure 1).

**Figure 2:**
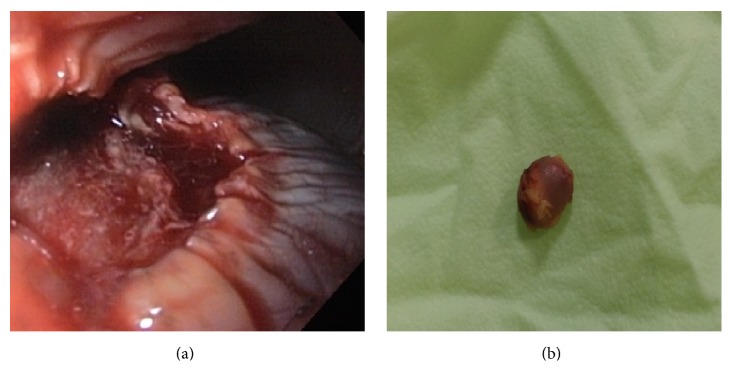
Endoscopic view of the esophagus after endoscopic treatment of tumor (a). Gross appearance of the tumor after endoscopic treatment (b).

The patient was considered for endoscopic resection of lesion. After deep sedation with intravenous Propofol (Lipuro-1%), submucosal injection of 5 ml diluted epinephrine solution (0.01 mg/ml) with 0.5 ml methylene blue was done using a sclerotherapy needle (ENDO-FLEX-Germany). After creation of the submucosal fluid bulging, the resection of lesion was performed using a monofilament rotatable polypectomy snare (Boston; 20 mm; output setting: cut 40 watts; coagulation 40 watts) without any complication (Figure 2). The resected specimen was removed by basket. The size of specimen was 12 × 5 mm with yellow color (Figure 2). The specimen was fixed in 10% formalin and transferred to the lab for histologic assessment.

**Figure 3:**
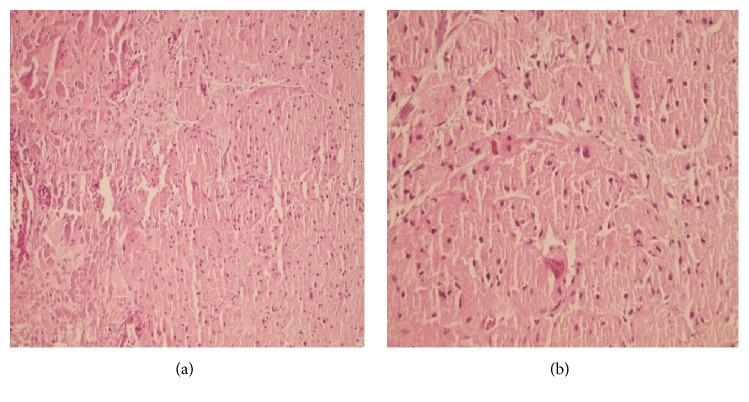
Section of the esophageal wall (a) showed nests of medium-sized cells with abundant eosinophilic granular cytoplasm (H&E ×100). High power view of the tumor in the esophageal wall (b) showed the above-mentioned cells with bland looking nuclei without any atypia or pleomorphism (H&E ×400).

In histologic examination, the diagnosis of GCT was confirmed. Histological analysis of specimen revealed nests of medium-sized cells with abundant eosinophilic granular cytoplasm with bland looking nuclei without any atypia or pleomorphism (Figure 3).

Follow-up endoscopy 9 months after removal of tumor showed complete healing of the site of resection in esophagus.

## 3. Discussion

The most common site of GCTs is tongue although these tumors may arise in other organs [2]. Gastrointestinal GCTs are uncommon. The most common site of gastrointestinal GCTs is esophagus that approximately 270 cases have been reported in the literature [4–6]. Esophageal GCTs usually appear as a white-gray to yellow submucosal mass that may be mistaken for other lesions such as lipoma, leiomyoma, and cyst [2, 4].

Esophageal GCTs are usually asymptomatic and found incidentally during endoscopy. The tumor may present with dysphagia when it is larger than 1 cm [2, 4, 7–9].

Although most esophageal GCTs have a benign clinical course, malignant tumors were reported in the literature. Malignant GCTs are usually larger than 4 cm [4, 10] with rapid growth and invasion of the adjacent tissues and high recurrence rate after excision [2, 11]. Tumor cell spindling and necrosis, increased mitotic activity with pleomorphism, and large nucleoli with high nuclear to cytoplasmic ratio are histologic evidence in favor of malignant GCTs [12].

EUS is useful technique for deciding the type of management of GCTs [4, 13]. EUS pattern of esophageal GCTs appears as a solid hypoechoic lesion with a smooth borders and mildly inhomogeneous echo pattern [2, 14]. They usually originate in the muscularis mucosa or submucosal layer of the esophagus. For evaluation of the role of EUS in the diagnosis of GCTs, a study in 15 patients with esophageal GCTs was performed by Palazzo et al. They concluded that EUS can contribute to planning the management of GCTs [14].

In order to compare EUS findings and histopathological analysis of resected submucosal lesions, Yazumi et al. studied 18 consecutive patients with esophageal submucosal tumors (5 GCTs and 13 leiomyomas). They concluded that EUS is useful for differentiating esophageal GCTs from leiomyomas [15].

The optimum management of GCTs is controversial [12]. Endoscopic follow-up for asymptomatic tumors less than 10 mm, endoscopic resection for tumors 10–20 mm [4, 12, 16, 17], and surgical excision for tumors more than 20 mm or symptomatic patient [12, 18] are generally recommended.

Similar to our case, Nakajima et al. reported a case of esophageal GCT diagnosed by endoscopic biopsy and EUS that treated by endoscopic submucosal dissection. They concluded that ESD is a safe and accurate procedure for GCTs [4]. De Ceglie et al. also reported a case of esophageal GCT diagnosed by endoscopic biopsy and EUS that was treated by EMR without any complication [2].

Similar to some reports, GCT in our patient was incidentally diagnosed by endoscopy [7, 9]. Because of the size of tumor 10–20 mm, similar to some reports, we treated the tumor by endoscopic resection [4, 16, 17].

Based on our experience and other published reports [2, 4, 14] we recommend that the submucosal lesion of esophagus can be evaluated by biopsy and EUS. If biopsy and/or EUS finding shows high possibility for diagnosis of GCT, endoscopic treatment can be safely scheduled for the tumors with size 10–20 mm.
